# Colonization of *Raphanus sativus* by human pathogenic microorganisms

**DOI:** 10.3389/fmicb.2024.1296372

**Published:** 2024-02-15

**Authors:** Sonia Szymańska, Edyta Deja-Sikora, Marcin Sikora, Katarzyna Niedojadło, Justyna Mazur, Katarzyna Hrynkiewicz

**Affiliations:** ^1^Department of Microbiology, Faculty of Biological and Veterinary Sciences, Nicolaus Copernicus University, Toruń, Poland; ^2^Center for Modern Interdisciplinary Technologies, Nicolaus Copernicus University, Toruń, Poland; ^3^Department of Cellular and Molecular Biology, Faculty of Biological and Veterinary Sciences, Nicolaus Copernicus University, Toruń, Poland

**Keywords:** human pathogenic microorganisms (HPMOs), Raphanus sativus, Escherichia coli, Salmonella enterica, Listeria monocytogenes, Bacillus cereus

## Abstract

Contamination of vegetables with human pathogenic microorganisms (HPMOs) is considered one of the most important problems in the food industry, as current nutritional guidelines include increased consumption of raw or minimally processed organic vegetables due to healthy lifestyle promotion. Vegetables are known to be potential vehicles for HPMOs and sources of disease outbreaks. In this study, we tested the susceptibility of radish (*Raphanus sativus*) to colonization by different HPMOs, including *Escherichia coli* PCM 2561, *Salmonella enterica* subsp. *enterica* PCM 2565, *Listeria monocytogenes* PCM 2191 and *Bacillus cereus* PCM 1948. We hypothesized that host plant roots containing bactericidal compounds are less prone to HPMO colonization than shoots and leaves. We also determined the effect of selected pathogens on radish growth to check host plant–microbe interactions. We found that one-week-old radish is susceptible to colonization by selected HPMOs, as the presence of the tested HPMOs was demonstrated in all organs of *R. sativus*. The differences were noticed 2 weeks after inoculation because *B. cereus* was most abundant in roots (log_10_ CFU – 2.54), *S. enterica* was observed exclusively in stems (log_10_ CFU – 3.15), and *L. monocytogenes* and *E. coli* were most abundant in leaves (log_10_ CFU – 4.80 and 3.23, respectively). The results suggest that *E. coli* and *L. monocytogenes* show a higher ability to colonize and move across the plant than *B. cereus* and *S. enterica*. Based on fluorescence *in situ* hybridization (FISH) and confocal laser scanning microscopy (CLSM) approach HPMOs were detected in extracellular matrix and in some individual cells of all analyzed organs. The presence of pathogens adversely affected the growth parameters of one-week-old *R. sativus,* especially leaf and stem fresh weight (decreased by 47–66 and 17–57%, respectively). In two-week-old plants, no reduction in plant biomass development was noted. This observation may result from plant adaptation to biotic stress caused by the presence of HPMOs, but confirmation of this assumption is needed. Among the investigated HPMOs, *L. monocytogenes* turned out to be the pathogen that most intensively colonized the aboveground part of *R. sativus* and at the same time negatively affected the largest number of radish growth parameters.

## Introduction

Vegetables are vital sources of carbohydrates, vitamins (e.g., B, C, K), minerals (e.g., calcium, potassium, and magnesium) and fiber. Consumption of raw plants (400 g per day) ensures health, prevents diseases (e.g., heart disease, cancer, diabetes and obesity) and is the basis of a balanced diet (Alemu et al., 2018; Balali et al., 2020). Current changes in nutritional guidelines resulting in increased demand for fresh or minimally processed vegetables have been linked to a higher incidence of foodborne illnesses in the past three decades (Mogren et al., 2018). Contamination of vegetables by human pathogenic microorganisms (HPMOs) can occur at the preharvest stage (e.g., soil amendment with improperly composted manure, soil irrigation with sewage or contaminated surface water, insects, dust or feces of wild animals) or during postharvest handling (e.g., washing, slicing, soaking, packing and food preparation) (Luna-Guevara et al., 2019; Jacob and Melotto, 2020). Plants can be colonized by HPMOs through natural openings, e.g., stomata, lenticels, hydathodes, sites of lateral root formation but also at sites of physical and chemical damage (Chahar et al., 2021). Raw food-borne HPMOs include *Escherichia coli* O157-H7 (Shiga toxin-producing bacteria), *Salmonella* spp., *Shigella* spp., *Listeria monocytogenes*, *Clostridium botulinum*, *Campylobacter* spp., *B. cereus*, *Aeromonas hydrophila*, *Vibrio cholerae*, and *Yersinia enterocolitica.*

The level of plant colonization by HPMOs can depend on many different aspects, e.g., differences in the metabolic traits of various plant species and genotypes, the stage of plant development and fruit maturity, the resistance of bacteria to antagonistic chemical compounds present in plant tissues and environmental conditions (Luna-Guevara et al., 2019; Jacob and Melotto, 2020). Notably, *E. coli*, *S. enterica* and *L. monocytogenes* are known to spread through contaminated water and soil directly to vegetables (Andino and Hanning, 2015; Carstens et al., 2019). HPMOs expelled from the host gut to the environment have different abilities to survive and multiply. *E. coli*, *L. monocytogenes* and *S. enterica* can persist in soil for different time periods, ranging from 3 days to 7–25 weeks or even a year, respectively (Winfield and Groisman, 2003; Miceli and Settanni, 2019). The colonization of plants by HPMOs is a complex process in which an important role is played by the ability of bacteria to attach to the plant surface, aggregate and/or form biofilms (Gorski et al., 2003; Jacob and Melotto, 2020). *E. coli* and *Salmonella* sp. can produce molecules important for the process of attachment and biofilm matrix formation in response to contact with plant leaves (Yaron and Römling, 2014). *L. monocytogenes* and *B. cereus,* due to their motility associated with flagellar presence and high potential to form biofilms, can efficiently colonize plant surfaces and tissues (Gorski et al., 2009; Majed et al., 2016; Lin et al., 2022). The mechanisms activated during plant invasion by specific HPMOs are still unclear; however, it was revealed that motile bacteria can more often colonize extracellular (apoplastic) spaces than the interior of plant cells (Holden et al., 2009). HPMOs show different specificities in the colonization of plant organs. Several works have confirmed the spread of pathogens in whole plants, while others have shown that pathogens behave like organ-specific microorganisms, e.g., colonizing only plant roots, irrespective of inoculation techniques and plant growth conditions (Cooley et al., 2003; Jablasone et al., 2005; Burris et al., 2020).

Based on genomic analysis six different subgroups of *E. coli* exist. They are able to colonize various ecological niches and can exhibit commensalistic or pathogenic lifestyle (van Elsas et al., 2011). Pathogenic *E. coli* can be responsible for numerous human infections associated with the consumption of contaminated vegetables. This microbe causes diarrhoeal disease of different severities, but cases of death are also noted (Mogren et al., 2018; Carstens et al., 2019). *E. coli* O157:H7 (infectious dose 50–100 cfu/g or mL) is one of the most common foodborne pathogens and contributes to several outbreaks (Sun et al., 2019; Puligundla and Lim, 2022), e.g., after consumption of romaine lettuce (58 persons/9 states) (Gupta and Madramootoo, 2017), baby spinach (26 states in the USA) (FDA, 2007), fresh washed spinach (2006: 199 cases/102 hospitalizations/3 deaths), ready-to-eat salads (2013: 33 cases/7 hospitalizations) and alfalfa sprouts (2016, 11cases/2 hospitalizations) and lettuce (Laven, 2006; Luna-Guevara et al., 2019).

According to the World Health Organization (WHO) report, the nontyphoidal *S. enterica* serotype is a common cause of foodborne diseases (*ca.* 79,000,000 cases in 2010) (Havelaar et al., 2015). Outbreaks of *S. enterica* have been related to different vegetables, such as romaine lettuce (2017, U.S. – 151 cases), onion (U.S., 2022–1,040 cases), raw almonds (2004, USA – 29 cases), black pepper (2010, USA – 272 cases), pistachios (2010, USA – 11 cases), iceberg lettuce (2008, Finland – 103/2 cases/deaths), baby spinach (2007, Sweden – 102 cases), tomatoes (2011, Denmark – 43 cases, Germany, Italy, Austria and Belgium – 28 cases), cucumber (2014, USA – 275/1 cases/death) and cantaloupe (2012, U.S. – 261/3 cases/deaths) (Denny et al., 2007; Lienemann et al., 2011; CDC, 2012; Müller et al., 2016; Clark, 2017; Dyda et al., 2020; Li et al., 2020; CDC, 2022).

*Listeria monocytogenes* has been found in various plant species, e.g., in sprouts of alfa-alfa, broccoli, cabbage, cantaloupe and radish. Microbe occurrence was noted both in high-income country, e.g., USA and Norway, and in developing countries, including India and Malaysia (Gorski et al., 2004, 2008, 2009; Ponniah et al., 2010; Walsh et al., 2014; Kljujev et al., 2018). The risk of getting ill from the consumption of contaminated vegetables is high because of the small infectious dose (ranging from 10^7^ to 10^9^ CFU in healthy hosts and 10^5^ to 10^7^ CFU in case of individuals at high risk of infection) (Farber et al., 1996). Several outbreaks were associated with the consumption of *L. monocytogenes*-contaminated: cantaloupe (2011, U.S. – 147/33 cases/deaths), sprouts (2014, U.S. – 5/2 cases/deaths), salad mix (2015–2016, U.S. – 19/1 cases/deaths) (CDC, 2012; Laksanalamai et al., 2012; CDC, 2016; Self et al., 2016; Gu et al., 2021).

*Bacillus cereus* includes highly versatile bacterial strains, inhabiting different environments including soil, plants, gut of healthy individuals and others (Ceuppens et al., 2013; Kulkova et al., 2022). They can posses various metabolic properties that predispose them to a pathogenic or commensal lifestyle. It has been noticed that specific strains of *B. cereus* can act as plant growth promoting bacteria having a beneficial influence on their hosts (Ceuppens et al., 2013; Kulkova et al., 2022). *B. cereus* comprise well-known food-borne pathogen; however, due to the sparse symptoms associated with infection, only a few reports exist (Dierick et al., 2005). The occurrence of *B. cereus* in fresh products reaches up to 37.5%, making this bacterium the most frequent foodborne pathogen (Kim et al., 2016). Analysis of different vegetables, including lettuce (Qu et al., 2021), onion, parsley, basil, coriander (Gdoura-Ben Amor et al., 2018), cucumbers, tomatoes, lettuce (Rosenquist et al., 2005; Yu et al., 2019), garlic chives, perilla leaf, romaine lettuce (Park et al., 2018), fresh peppers, carrots, zucchini, garlic (Valero et al., 2002), and broccoli (Flores-Urban et al., 2014), collected from China (Yu et al., 2019), Korea (Park et al., 2018), Tunisia (Gdoura-Ben Amor et al., 2018), and Mexico (Flores-Urban et al., 2014), confirmed the presence of *B. cereus*. *B. cereus*-related food poisoning outbreaks are estimated to be approximately 3.9–5.9% (reports for 2011–2015), some of which have ben related to the consumption of vegetables such as tomatoes (2007, France, 4 cases), potatoes (2008, France, 28 cases), salad (2010, France, 44 cases) and carrots (2011, France, 3 cases) (Glasset et al., 2016; Jessberger et al., 2018).

Existing studies have shown that many vegetables are efficiently colonized by HPMOs. However, there are reports that some plants, e.g., cauliflower, broccoli, and okara byproducts, have antimicrobial potential against different food-borne pathogens, including *S. enterica* serovar Typhimurium, *E. coli* O157: H7 and *B. cereus* (Sanz-Puig et al., 2015). In the case of *L. monocytogenes*, similar observations were made for tomatoes and carrots (Gorski et al., 2003). Radish (annual herb, member of Brassicaceae, commonly consumed by humans) may belong to the group of vegetables less susceptible to colonization by HPMOs. In 1947, it was found that raphanin contained in radish seeds has bacteriostatic activity against different bacteria (*Staphylococcus* and *E. coli*) (former *Bact. coli*) (Ivánovics and Horváth, 1947). Over the next years, knowledge about the antibacterial compounds contained in radish seeds was extended. The following pathogens, i.e., *Streptococcus pyogenes*, *E. coli*, *Salmonella enteritidis* 110, *Cronobacter sakazakii* KCTC 2949, *B. cereus* ATCC 10876 and *Staphylococcus aureus* ATCC 6538 are sensitive to radish metabolites (Jadoun et al., 2016; Lim et al., 2019). The effect of red-peel radish root extract on 52 food-borne bacteria confirmed the presence of compounds with antimicrobial activity (Jadoun et al., 2016; Kaymak et al., 2018). The authors emphasized the potential role of these compounds in preventing microbial contamination of food (Kaymak et al., 2018).

The main aim of our research was to determine the interactions between radish and selected species of HPMOs: *E. coli* PCM 2561, *S. enterica* subsp. *enterica* PCM 2565, *L. monocytogenes* PCM 2191 and *B. cereus* PCM 1948. We hypothesized that (i) radish roots, rich in bactericidal substances, would be the plant organ least susceptible to colonization by the tested food-borne pathogens compared to leaves and shoots and (ii) HPMOs would have a negative effect on plant growth. In this study, we used one-week- and two-week-old radish organs (roots, stems and leaves) to examine their colonization level by the tested species with culture-dependent plating and culture-independent qPCR methods. Using the FISH technique, we also determined *in situ* the colonization of bacteria in the organs of all variants of plant cultivation. Additionally, the impact of pathogens on plant growth parameters (root, aboveground plant and plant length, fresh and dry weight of leaves, stem, root and whole plant) was investigated.

## Materials and methods

### Experimental design

**Figure 1:**
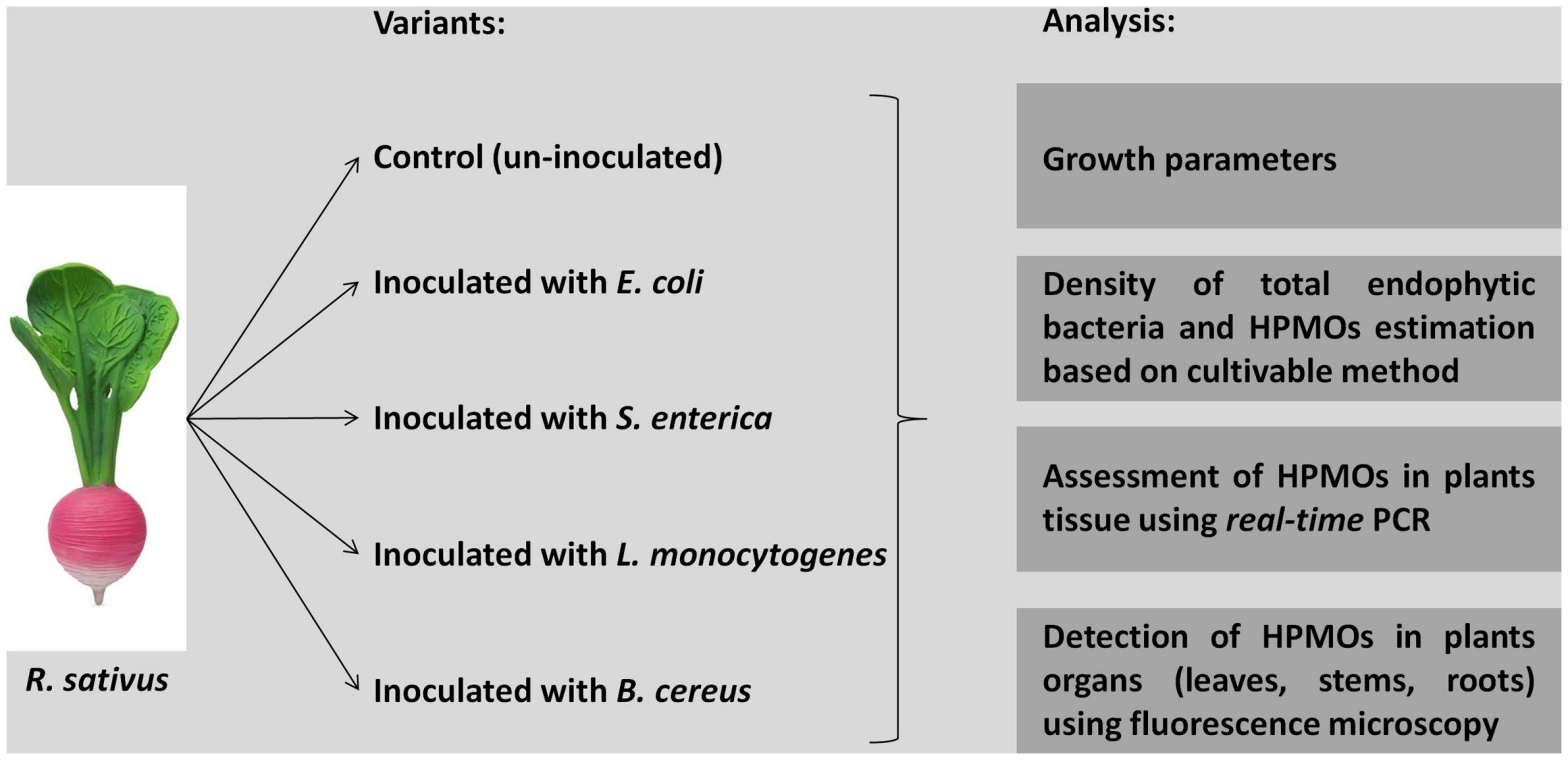
Experimental design.

*Raphanus sativus* seeds were obtained from Plantico Company. Healthy and uniform-sized seeds were surface sterilized with 70% ethanol for 2 min, rinsed three times with sterile distilled water, treated with 30% sodium hypochlorite for 2 min and washed three times with sterile distilled water. The liquid obtained from the last washing was used to assess the efficiency of sterilization by plating on R2A (Difco) and Martin (BTL) agar media. Bacterial strains, i.e., *E. coli* Group A PCM 2561, *S. enterica* subsp. *enterica* serovar Choleraesuis PCM 2565, *L. monocytogenes* serovar 01/2 PCM 2191 and *B. cereus* PCM 1948, were cultivated in liquid trypticase soy broth (TSB, BD) with shaking at 37°C for 24 h. Then, suspensions containing 1.5 × 10^8^ cells/ml (equivalent to McFarland OD = 0.5) were prepared. Surface-sterilized seeds were incubated in suspensions of the respective HPMO strains for 45 min with shaking (120 rpm) (Figure 1). As a control, sterilized distilled water was used instead of bacterial suspension. Treated seeds were cultivated in MS medium (Duchefa) in plastic rectangular shaped containers (107 × 94 × 96 mm, Duchefa, 10 plants/container, 10 containers/one variant) under controlled environmental conditions (temperature: 22°C ± 2°C and light/dark: 16 h/8 h). After 1 and 2 weeks, plants were carefully removed from the containers, surface sterilized with 70% ethanol for 2 min, rinsed with sterile distilled water and dried on sterile tissue paper. Collected plants were analyzed as shown in Figure 1.

### Plant growth parameters analysis

Different growth parameters were analyzed, including total plant length, shoot and root length, fresh and dry weight (72 h drying at 85°C) of leaves, stems, roots and plants.

### Estimation of total endophytic bacteria and selected HPMOs density in different organs of *Raphanus sativus*

Separated plant organs (leaves, stems or roots; five biological replicates per variant) were homogenized using a mortar and pestle with sterile distilled water in a 1:9 ratio (sample weight:volume of sterile distilled water). Serial dilutions (10^−1^ −10^−3^) of all homogenates were plated in triplicate on trypticase soy agar (TSA, BD) and appropriate selective media for *S. enterica* (Chromogenic, *Salmonella* LAB-AGAR TM and *Salmonella* Chromogenic Supplement, Biomaxima), *E. coli* (*E. coli* Chromogenic Medium, Biomaxima), *B. cereus* (*B. cereus* Selective LAB – AGAR^™^ Base and *B. cereus* Supplement, Biomaxima), and *L. monocytogenes* (Chromogenic *Listeria* acc. to Ottaviani and Agostii LAB -AGAR^™^ Base and Chromogenic Listeria Supplement acc. to ISO 11290, Biomaxima). Prepared plates were incubated for two (selective media) and three (TSA medium) days at 37°C. Colonies were counted each day. After 24 h, all colonies of *E. coli*, *B. cereus* and *L. monocytogenes* were fully grown, while the highest density of *S. enterica* was observed after two or three (in the case of TSA medium) days of cultivation. Colony forming units (CFU) were counted and calculated per g of fresh plant weight using plates with 30–300 colonies.

### Assessment of HPMOs in plant tissue using *real-time* PCR

#### Plant material collection and genomic DNA isolation

Surface sterilized roots, stems and leaves were packed separately in 1.5 mL Eppendorf tubes and stored at −80°C. Three biological replicates were prepared per plant organ: roots, stems and leaves (nine samples per variant were obtained in total). Genomic DNA isolation was performed using a Plant and Fungi DNA Purification Kit (EURx, Poland) following the manufacturer’s procedure with a modified homogenization step (FastPrep-24 bead-beater, three cycles of 20 s at 4.5 m/s). DNA was quantified using a Qubit™ dsDNA HS Assay Kit (Invitrogen^™^). DNA was stored at −80°C.

#### *Real-time* quantitative PCR assay

Absolute quantifications of each investigated HPMO (due to lack of specific primers for *B. cereus* this microorganism was not tested with qPCR) and total 16S rRNA gene copy (expressed as pEF copy number) determination in roots, stems and leaves of *R. sativus* were carried out using LightCycler 480 and LightCycler 480 SYBR Green I Master Kit (Roche). qPCR was performed in a total volume of 10 μL, which contained 5 μL of 2x SYBR Green I Master Mix (Roche), 3.5 μL of H_2_O, 0.25 μL of each specific primer (10 pmol/μl, Table 1) and 1 μL of DNA template (20 ng/μl for stems and leaves and 6 ng/μl for roots). The cycling conditions included initial denaturation for 5 min at 95°C, then 40 cycles of 10 s of denaturation at 95°C, 20 s of annealing at the optimal temperature for the primer pair (Table 1), 20 s of elongation at 72°C, and finally a melting curve with a continuous temperature increase from 65°C to 95°C. Positive (DNA of specific strains) and negative (molecular grade water) controls were analyzed in parallel with experimental samples. All qPCR analyses were performed in three biological and technical replicates (nine results for each variant were obtained).

**Table 1:** Oligonucleotide sequences used in *q*PCR.

Oligo name	Sequence (5 → 3)	Detected bacteria	Annealing temperature	Reference
EcRTF1	GAAGGGAGTAAAGTTAATAC	*E. coli*	57	This study
EcRTR1	AGTATCAGATGCAGTTCC	*E. coli*		This study
SeRTF1	TGGTCTGAGAGGATGCCAG	*S. enterica*	59	This study
SeRTR1	GCGGTTATTAACCACACACC	*S. enterica*		This study
47F	GTG ACA AAT GTG CCG CCA AG	*L. monocytogenes*	63	This study
892R	TCC GAG GTT ACC GTC GAT GA	*L. monocytogenes*		This study
S-D-Bact-0907-a-S-20	ACG AGC TGA CGA CAG CCA TG	Total bacteria	59	ProbeBase
S-D-Bact-1054-a-A-20	AAA CTC AAA GGA ATT GAC GG	Total bacteria		ProbeBase

Standard curves for quantifications of 16S rDNA copy number for each strain were prepared based on amplicons generated with 27F (5′AGA GTT TGA TCM TGG CTC AG 3′) and 1492R (5′ TAC GGY TAC CTT GTT ACG AC 3′) universal primers (Lane, 1991). PCRs were prepared using 2x Plus Taq Mix (Qiagen). Annealing conditions for the primer pair used in PCR included incubation for 30 s at 50°C. Amplicons were purified with AMPure XP (Beckman Coulter) and checked for their concentration with a Qubit^™^ dsDNA HS Assay Kit (Invitrogen^™^, USA) and Qubit fluorometer (ThermoFisher Scientific, USA). Standard curves were prepared using 10-fold dilutions of amplicons containing 10 million, 1 million, 100,000, 10,000, 1,000, and 100 copies per reaction. Based on the standard curve, the reaction efficiency was determined for each primer set. The results of absolute quantification were expressed as copies of specific bacterial 16S rRNA genes in 1 ng of total plant DNA. To determine the fold change in HPMO quantity in plant organs, the values were then calculated against the control samples. Furthermore, the percentage of HPMOs was calculated in relation to the total copy number of bacterial 16S rRNA genes.

### FISH detection of HPMOs in plant organs

The roots, stems and leaves of *R. sativus* seedlings (three per variant) were fixed in 4% paraformaldehyde (Polyscience, USA) and 0.25% glutaraldehyde (Sigma-Aldrich, USA) in phosphate-buffered saline (PBS) buffer pH 7.2 overnight at 4°C and after dehydration were embedded in BMM resin (butyl methacrylate, methyl methacrylate, 0.5% benzoyl ethyl ether) (Sigma-Aldrich, USA) with 10 mM DDT (Thermo Fisher Scientific, USA) according to Niedojadło et al. (2015). The material was cut on a Leica UCT ultramicrotome into serial cross semithin sections and collected on Thermo Scientific^™^ Polysine (Thermo Fisher Scientific, USA) adhesion microscope slides. Before FISH reaction, the resin was removed with two changes of acetone and washed in distilled water and 4xSSC (Saline-Sodium Citrate buffer, Sigma-Aldrich, USA) and incubated with lysozyme 1 mg/mL (Thermo Fisher Scientific, USA). A *E. coli*, *L. monocytogenes* and *S. enterica* specific sequences targeting the 16S rRNA were used as specific FISH probes (Genomed, Poland) and were resuspended in hybridization buffer (Sigma-Aldrich, USA) with 30% *v/v* formamide at a concentration of 50 pmoL/mL. The following antisense DNA oligonucleotides labeled with Cy3 were used for the reactions for the detection of: *E. coli* 5′-Cy3-ACATCCGATGGCAAGAGGCCCGAA GGT-3′, *L. monocytogenes* 5′-Cy3-CGATAGCCGAAACCATCTTTCA AAAGCGTGG-3′, *S. enterica* 5′-GCTGCGGTTATTAACCACAAC ACCTTCCTC-3′. Hybridization was performed overnight at 42°C in a humidified chamber. After reaction the material was stained for DNA detection with Hoechst 33342 (1,1,000) (Invitrogen, USA) and mounted in ProLong Gold Antifade reagent (Invitrogen, USA). The positive control reactions were performed using only embedded bacteria. Two types of the negative control reactions were carried out: the samples were incubated only with hybridization buffer or reaction was performed on the samples from an uninoculated *R. sativus* seedling.

The images were captured with Olympus FV3000 confocal laser scanning microscope (CLSM). The optimized pinhole, long exposure (400 Hz), and 63X (numerical aperture 1.4) Plan Apochromat DIC H oil immersion lens were used. The images were collected simultaneously in the blue (Hoechst 33342) and red (Cy3) channels. To minimize bleed-through between the channels, we employed low laser power (1.5% of maximum power) and sequential collection. For all probes and plants organs, the obtained data were corrected for background autofluorescence, as determined by negative-control signal intensities. All image acquisition was performed using constant parameters (laser power, detector gain, emission band, and resolution).

### Statistical analyses

One-way ANOVA and Newman–Keuls *post hoc* tests were used to compare growth parameters (including fresh and dry weight of leaves, stems, roots, plant, as well as length of roots and shoots) and colonization level (total density of endophytes and HPMOs in leaves, stems and roots) between treatments (i.e., uninoculated *R. sativus –* Ctr and plants inoculated with HPMOs: *L. monocytogenes* PCM 2191/L.m., *B. cereus* PCM 1948/B.c., *E. coli* PCM 2561/E.c. and *S. enterica* subsp. *enterica* PCM 2565/S.e.) depending on the cultivation time (1 or 2 weeks). Calculations were performed with the Statistica 10.0 software package (StatSoft, 2006).

The effect of treatment (Ctr, L.m., B.c., E.c, S.e.) on the growth parameters of *R. sativus* and the rate of its colonization by endophytes and HPMOs was checked with multivariate statistical analysis including principal component analysis (PCA) (R software). The analyses were performed for samples collected after the first and the second week of the experiment. For each PCA, the statistical significance was checked using the ‘arleyc/PCAtest’ R package. Statistically significant results were used to prepare the PCA plot.

## Results

### HPMOs affect the growth of *Raphanus sativus*

**Figure 2:**
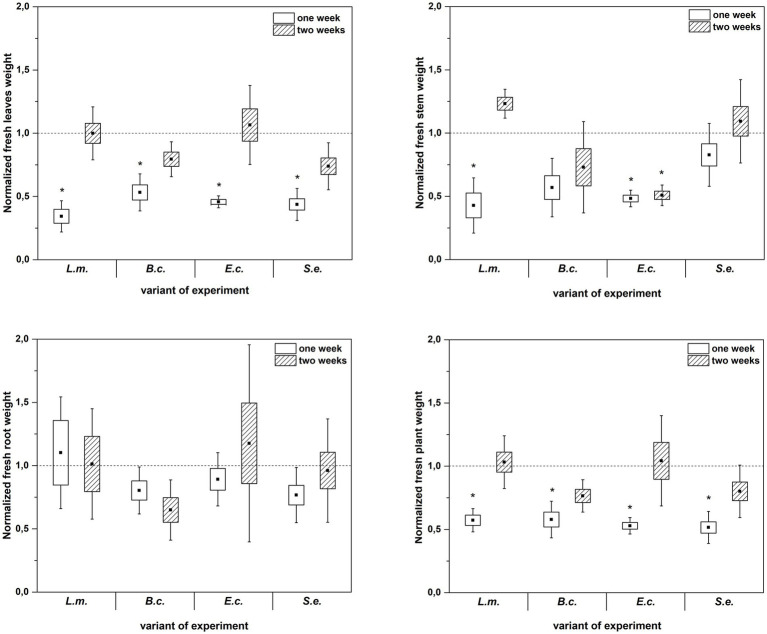
Normalized fresh leaves, stems, roots and plant weight of *R. sativus* inoculated with *L. monocytogenes* PCM 2191 – L.m., *B. cereus* PCM 1948 – B.c., *E. coli* PCM 2561 – E.c. and *S. enterica* subsp. *enterica* PCM 2565 – S.e. after 1 and 2 weeks of plant cultivation. Significant differences (*p* < 0.05, one-way ANOVA with Newman–Keuls *post hoc* comparisons) between the control (noninoculated) and inoculated variants at each time of *R. sativus* cultivation are denoted by different marks (*).

**Figure 3:**
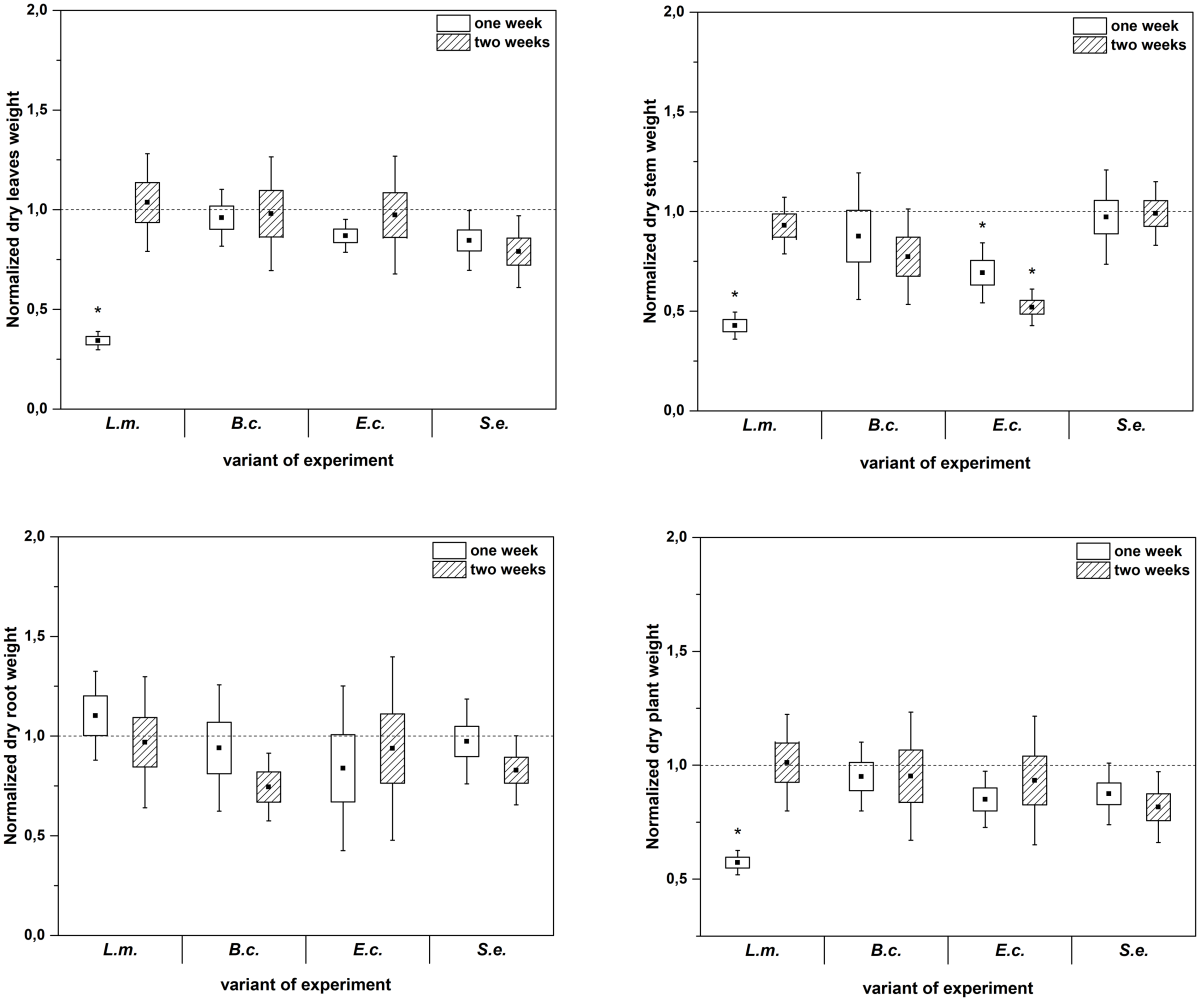
Normalized dry leaves, stem, root and plant weight of *R. sativus* inoculated with *L. monocytogenes* PCM 2191 – L.m., *B. cereus* PCM 1948 – B.c., *E. coli* PCM 2561 – E.c. and *S. enterica* subsp. *enterica* PCM 2565 – S.e. after 1 and 2 weeks of plant cultivation. Significant differences (*p* < 0.05, one-way ANOVA with Newman–Keuls *post hoc* comparisons) between the control (noninoculated) and inoculated variants at each time of *R. sativus* cultivation are denoted by different marks (*).

**Figure 4:**
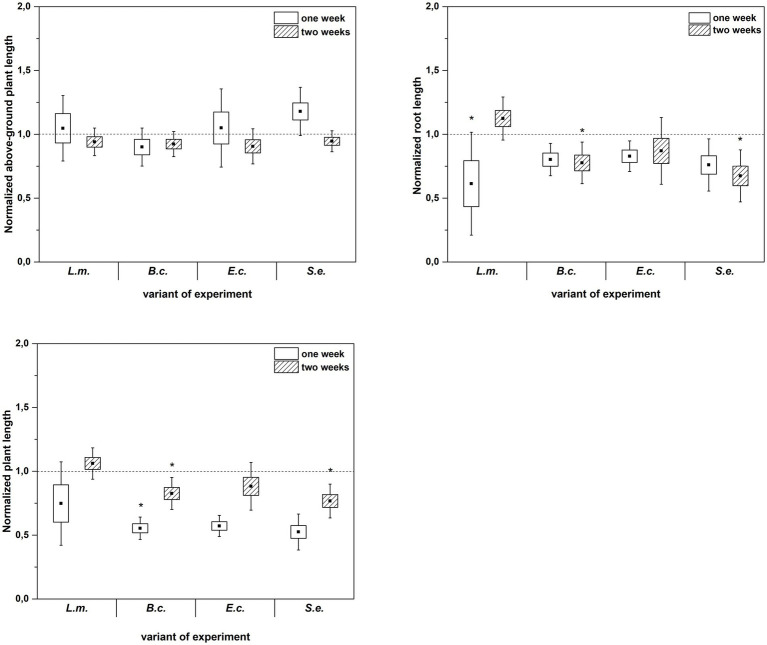
Normalized aboveground plant length, root length and plant length of *R. sativus* inoculated with *L. monocytogenes* PCM 2191 – L.m., *B. cereus* PCM 1948 – B.c., *E. coli* PCM 2561 – E.c. and *S. enterica* subsp. *enterica* PCM 2565 – S.e. after 1 and 2 weeks of plant cultivation. Significant differences (*p* < 0.05, one-way ANOVA with Newman–Keuls *post hoc* comparisons) between the control (noninoculated) and inoculated variants at each time of *R. sativus* cultivation are denoted by different marks (*).

Inoculation of *R. sativus* with HPMOs (L.m., B.c., E.c, S.e.) negatively affected the investigated plant growth parameters; however, the level of impact depended on the plant age (1 or 2 weeks) and the species of pathogen (Figures 2–4). Changes in radish growth parameters in response to the presence of specific bacterial strain were more evident in one-week-old seedlings than in two-week-old seedlings (Figures 2–4). One-week-old seedlings upon inoculation were characterized by both lower total fresh biomass and decreased fresh weight of leaves compared to the control (Ctr, noninoculated plants) (Figure 2). Among the tested HPMOs, *L. monocytogenes* influenced the highest number of growth parameters in one-week-old radish, including root length (decreased by 39% compared to the control variant), stem fresh weight (reduced by 57%) and dry weight of leaves, stem and whole plant (decreased by 66, 57 and 43%, respectively) (Figures 2–4). The remaining strains negatively affected only some growth parameters, e.g., *B. cereus* reduced plant length (by 45%), while *E. coli* decreased stem fresh (by 51%) and dry (by 31%) weight (Figures 2–4). In the case of two-week-old plants, *S. enterica* and *B. cereus* decreased the length of whole plants (23 and 17%) and roots (33 and 22%), and *E. coli* reduced stem fresh (49%) and dry (48%) weight (Figures 2–4).

### Abundance of endophytes and HPMOs in plant tissues of *Raphanus sativus*: cultivation-dependent and cultivation-independent methods

Cultivation-dependent methods revealed that the total numbers of endophytes and HPMOs in the studied *R. sativus* organs (roots – R, stems – S and leaves – L) were associated with the plant age and the bacterial strain chosen for inoculation (Supplementary Figure S1 and Tables 2, 3).

**Table 2:** Total density of endophytic bacteria in leaves, stems and roots of *R. sativus* for control (Ctr, uninoculated plants) and variants inoculated with *L. monocytogenes*, *B. cereus*, *E. coli* and *S. enterica* after 1 and 2 weeks of cultivation.

Total density of endophytes	Organ	Ctr	*L. monocytogenes*	*B. cereus*	*E. coli*	*S. enterica*
1 week old seedlings	Leaves	2.9134 (0.085) a	3.2486 (0.355) a	2.8216 (0.230) a	3.9860 (0.070) b	4.2157 (0.283) b
	Stems	3.3117 (0.004) b	2.8968 (0.114) a	2.8503 (0.062) a	3.5997 (0.751) b	4.5158 (0.034) b
	Roots	4.6608 (0.030) c	3.7884 (0.091) b	4.4771 (0.000) b	2.6278 (0.086) a	2.7558 (0.145) a
2 week old seedlings	Leaves	4.0012 (0.1351) b	5.1304 (0.0487) c	1.2594 (0.2413) a	5.3859 (0.0518) c	3.8690 (0.1534) b
	Stems	3.7128 (0.0906) a	4.1135 (0.0231) b	2.1150 (0.0560) b	3.4370 (0.1185) a	4.6952 (0.0175) c
	Roots	3.7321 (0.0210) a	1.6414 (0.2967) a	2.5820 (0.1816) c	3.9635 (0.0189) b	1.4337 (0.2298) a

Significant differences (based on *p* < 0.05, one-way ANOVA with Newman–Keuls *post hoc* comparisons) between organs for each cultivation time (1 and 2 weeks) are denoted by different letters. Mean values of log_10_ CFU/g dry weight and standard deviation are presented (*n* = 3).

**Table 3:** Density of HPMO in leaves, stems and roots of *R. sativus* for variants inoculated with *L. monocytogenes*, *B. cereus*, *E. coli* and *S. enterica* after 1 and 2 weeks of cultivation.

Density of HPMO	Organs	*L. monocytogenes*	*B. cereus*	*E. coli*	*S. enterica*
1 week old seedlings	Leaves	4.2605 (0.0395) c	2.8189 (0.0299) c	3.4182 (0.1118) a	1.2007 (0.1738) b
	Stems	3.0785 (0.0290) b	2.5676 (0.1049) b	3.891 (0.0461) c	3.0352 (0.1974) c
	Roots	1.2007 (0.1738) a	0.94837 (0.0894) a	3.6353 (0.0810) b	0.5773 (0.5774) a
2 weeks old seedlings	Leaves	4.8055 (0.0737) c	0.0000 (0.0000) a	3.2275 (0.0625) c	0.0000 (0.0000) a
	Stems	3.7984 (0.0661) b	2.0473 (0.0961) b	2.7940 (0.1136) b	3.1491 (0.2654) b
	Roots	0.0000 (0.0000) a	2.5396 (0.1085) c	0.0000 (0.0000) a	0.0000 (0.0000) a

Significant differences (based on *p* < 0.05, one-way ANOVA with Newman–Keuls *post hoc* comparisons) between organs for each cultivation time (1 and 2 weeks) are denoted by different letters. Mean values of log_10_ CFU/g dry weight are presented (*n* = 3).

The time of plant cultivation significantly affected the total abundance of endophytes. Higher numbers of endophytes were noted in the roots of one-week-old *R. sativus* in almost all variants (with the exception of plants inoculated with *E. coli*) compared to stems and leaves. The aboveground parts of *R. sativus* were more intensively colonized after 2 weeks of cultivation, with the exception of leaves of plants treated with *S. enterica* and stems of plants inoculated with *E. coli*, where a similar total density of endophytic bacteria was observed (Supplementary Figures S1A–C and Table 2).

The inoculation of plants with HPMOs resulted in a decrease in the total density of endophytes in the roots of one-week- and two-week-old *R. sativus* compared to the noninoculated control variant (for L.m.: 18.7% vs. 56.0%, B.c.: 3.9% vs. 30.8%, S.e: 40.9% vs. 61.6%). Interestingly, in the case of *E. coli,* the amount of bacteria was similar regardless of the cultivation time (Supplementary Figure S1C and Table 2). In stems and leaves of one-week-old plants, a similar density of endophytes was noted for all tested variants with the exception of *S. enterica*-treated plants (increases of 36.4 and 44.7% were observed in stems and leaves, respectively). The presence of HPMOs significantly changed the total density of endophytes in the aboveground parts of two-week-old plants. The number of endophytes in stems and leaves increased in L.m. variant (by 10.8 and 28.2%, respectively). Opposite observations were made for the B.c. variant, where a decrease in the abundance of bacteria was noted (by 43.1% in stems and 68.5% in leaves) (Supplementary Figures S1A,B and Table 2).

HPMOs were found in all organs of one-week-old plants, while in the case of two-week-old *R. sativus*, no pathogens were detected in specific organs. The absence of L.m., E.c. and S.e. was observed in roots. Surprisingly, the leaves of plants inoculated with B.c. and S.e. were pathogen-free (Supplementary Figures S1D–F and Table 3). In general, HPMOs (excluding B.c.) more efficiently colonized the aboveground parts of plants, regardless of their age. B.c. preferentially inhabited the roots of two-week-old plants (Supplementary Figures S1D–F and Table 3). *L. monocytogenes* showed the highest ability to colonize *R. sativus* leaves at both cultivation times and stems of two-week-old plants compared to other tested HPMOs (Supplementary Figures S1D,E and Table 3).

**Figure 5:**
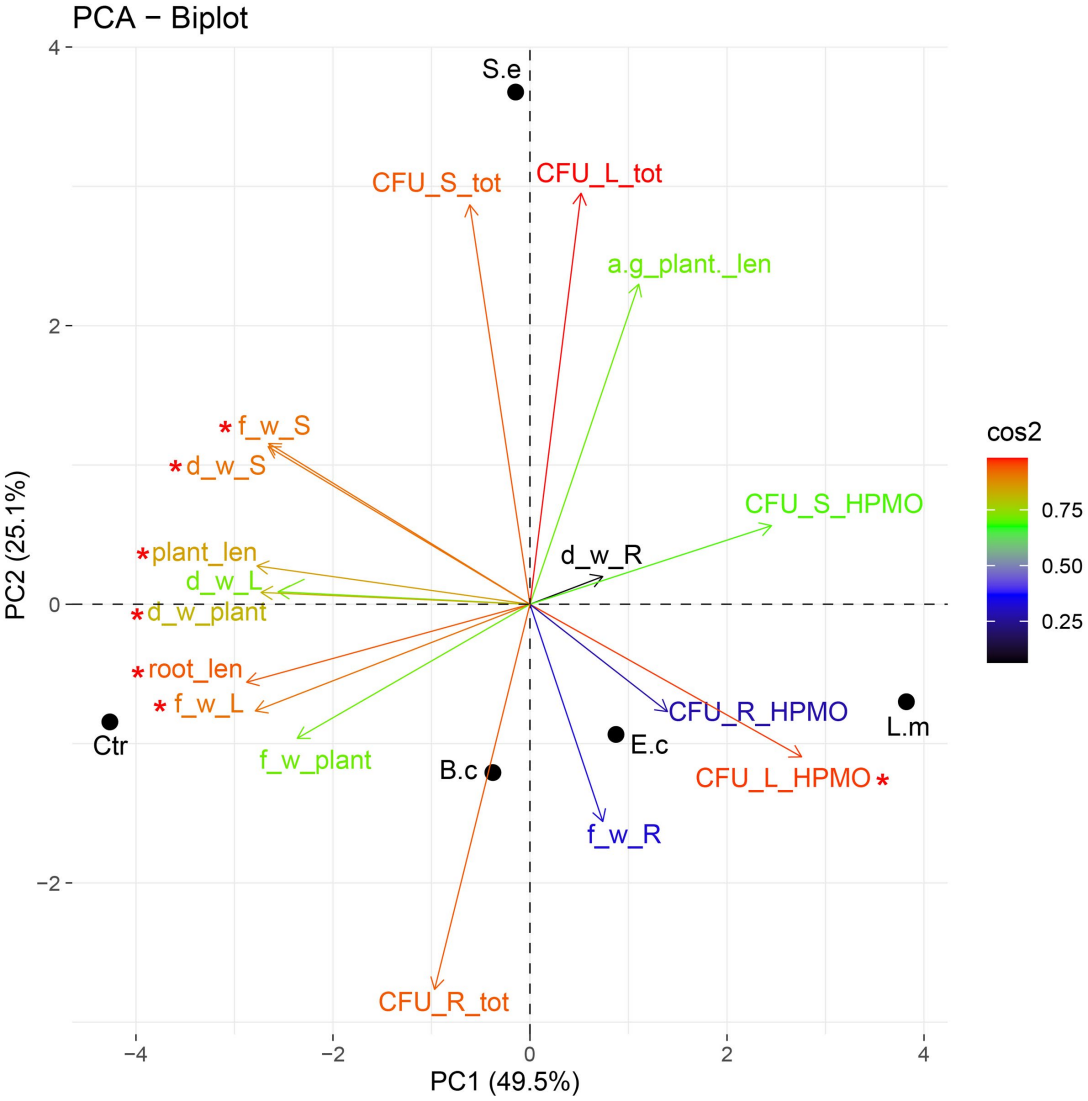
PCA ordination of growth parameters and density of endophytes after one **(A)** and two **(B)** weeks of *R. sativus* cultivation. a-g_plant _len, aboveground plant length; root_len, root length; plant_len, plant length; f_w_L, fresh leaf weight; f_w_R, fresh root weight; f_w_S, fresh stem weight; f_w_plant, fresh plant weight; d_w_L, dry leaf weight; d_w_R, dry root weight; d_w_S, dry stem weight; d_w_plant, dry plant weight; CFU_L_tot, total density of endophytes in leaves; CFU_S_tot, total density of endophytes in stems; CFU_R_tot, total density of endophytes in roots; CFU_L_HPMO, density of HPMOs in leaves; CFU_S_HPMO, density of HPMOs in stems; CFU_R_HPMO density of HPMOs in roots.

Principal component analysis (PCA) showed a dispersion of the investigated variants in the ordination space of PC1-PC2. The PC1 and PC2 axes explained nearly 75% (49.52 and 25.10%, respectively) of the total variance. PC1 was significant (*p* value 0.01) in explaining the variance between the samples. The variables having significant loadings on PC1 included root and whole plant length, leaf and stem fresh weight and HPMO content in leaves (all parameters are marked in the PCA plot with asterisks). The inoculation with *L. monocytogenes* and *E. coli* exerted a similar effect on one-week-old plants, as both were associated (positively correlated) with root fresh weight and elevated HPMO density in leaves and to a lesser extent in roots (Figure 5). The impact of *S. enterica* was different because a negative correlation of this pathogen with all tested parameters was observed (Figure 5). Ordination analysis for samples collected after the second week was not statistically significant.

**Figure 6:**
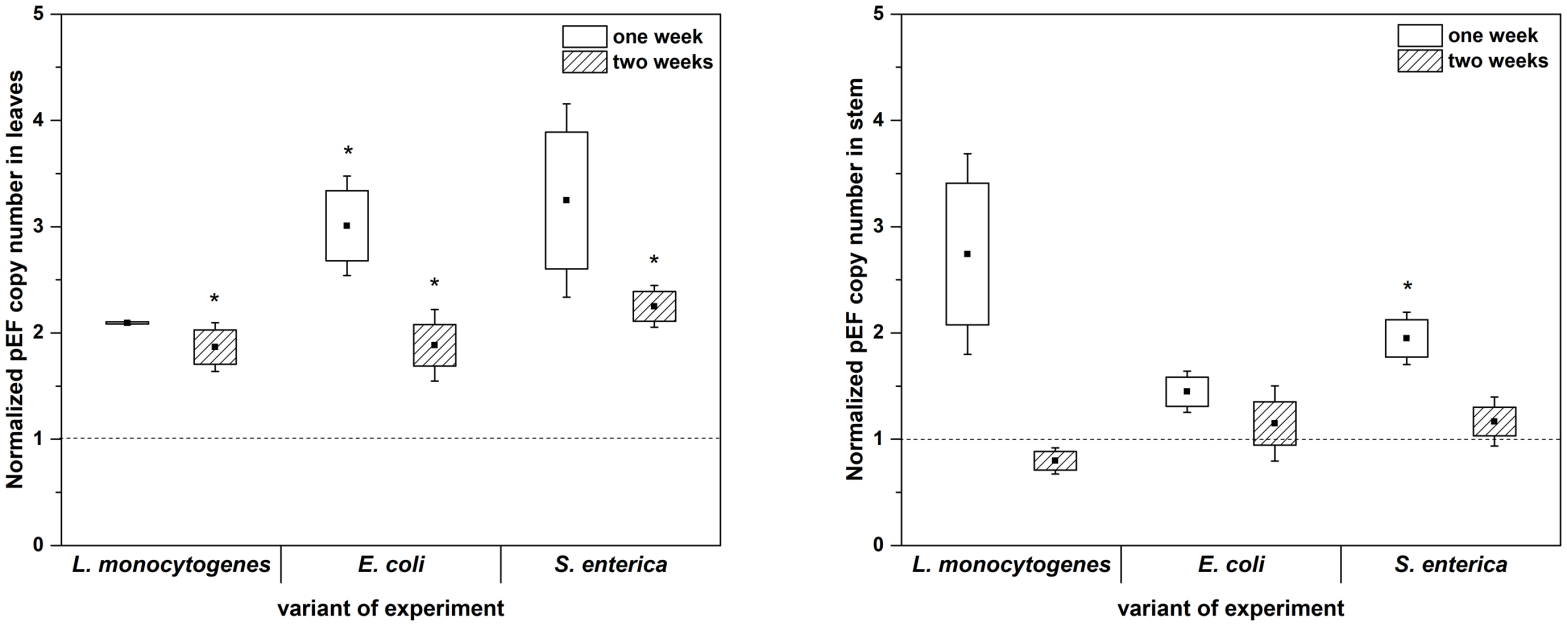
Normalized pEF copy number in leaves and stems of *R. sativus* after 1 and 2 weeks of plant cultivation. Significant differences (*p* < 0.05, one-way ANOVA with Newman–Keuls *post hoc* comparisons) between control and inoculated (inoculated with *L. monocytogenes* PCM 2191, *E. coli* PCM 2561 and *S. enterica* subsp. *enterica* PCM 2565) treatments are denoted by different marks (*).

The culture-independent method (qPCR analysis) partially confirmed the results obtained with the use of microbiological media. In this assay, inoculation with *L. monocytogenes* also showed no effect on the total 16S rRNA gene copy number in either the first or the second week of plant cultivation (with the exception of two-week-old plants leaves). Similarly, inoculation with *E. coli* caused an increase in the total 16S rRNA gene copy number in leaves of two-week-old *R. sativus* compared to the uninoculated variant (Figure 6). Only the presence of *S. enterica* was associated with a significantly increased 16S rRNA gene copy number in stems of one-week-old plants (Figure 6). Contrary to the results based on cultivation, qPCR showed no effect of inoculation with tested strains on 16S rRNA gene copy number in stems of two-week-old *R. sativus*. Additionally, inoculation with *S. enterica* did not influence the 16S rRNA gene copy number in leaves of one-week-old plants compared to control variants, but an increase in that number was noticed after 2 weeks (Figure 6).

**Figure 7:**
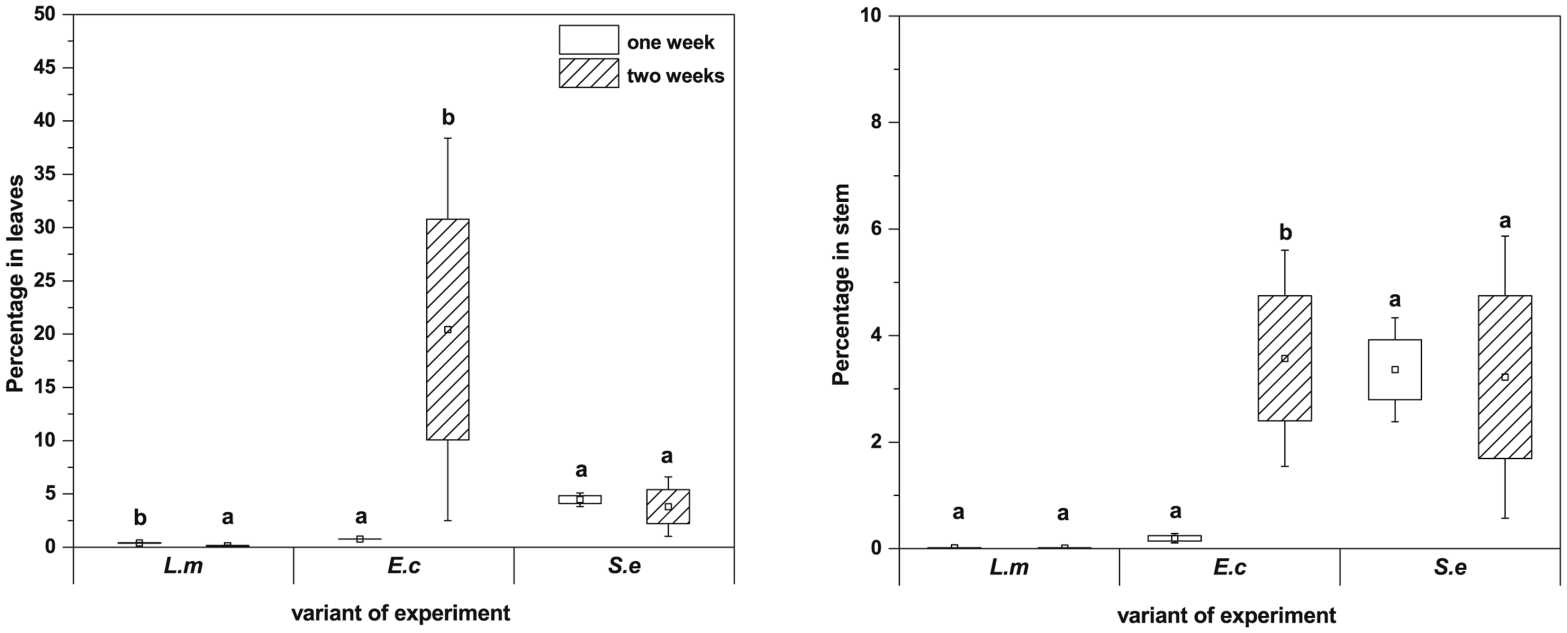
Significant differences (*p* < 0.05, one-way ANOVA with Newman–Keuls *post hoc* comparisons) between percentage share of HPMO (*L. monocytogenes*, *E. coli*, *S. enterica*) in stem and leaves of *R. sativus* obtained for two tested times of plant cultivation (1 and 2 weeks). The presented values were calculated in relation to total density of bacteria (based on pEF gene copy number) amounting 100%. Standard error (box) and standard deviation (whiskars) are shown.

qPCR analysis showed a higher proportion of *L. monocytogenes* and *E. coli* in relation to the total number of bacteria in plant leaves (1 week: L.m. – 0.41% and E.c. – 0.77%; 2 weeks: L.m. – 0.75% and E.c. – 20.43%) compared to stems (1 week: L.m. – 0.02% and E.c. – 0.24%; 2 weeks: L.m. – 0.01% and E.c. – 3.57%) (Figure 7). The distribution of *S. enterica* in leaves (1 week: 4.46% and 2 weeks: 3.80%) and stems (1 week: 3.36% and 2 weeks: 4.31%) was similar in both tested cultivation variants (1 and 2 weeks) (Figure 7). The relative level of *L. monocytogenes* in leaves and stems was the lowest in comparison to the other investigated pathogens. Based on the qPCR results, *E. coli* had the highest ability to colonize leaves of two-week-old *R. sativus*, while *S. enterica* preferentially colonized the stems of both one-week-old and two-week-old plants (Figure 7).

### Visualization of HPMOs colonization in plant organs of *Raphanus sativus*

**Figure 8:**
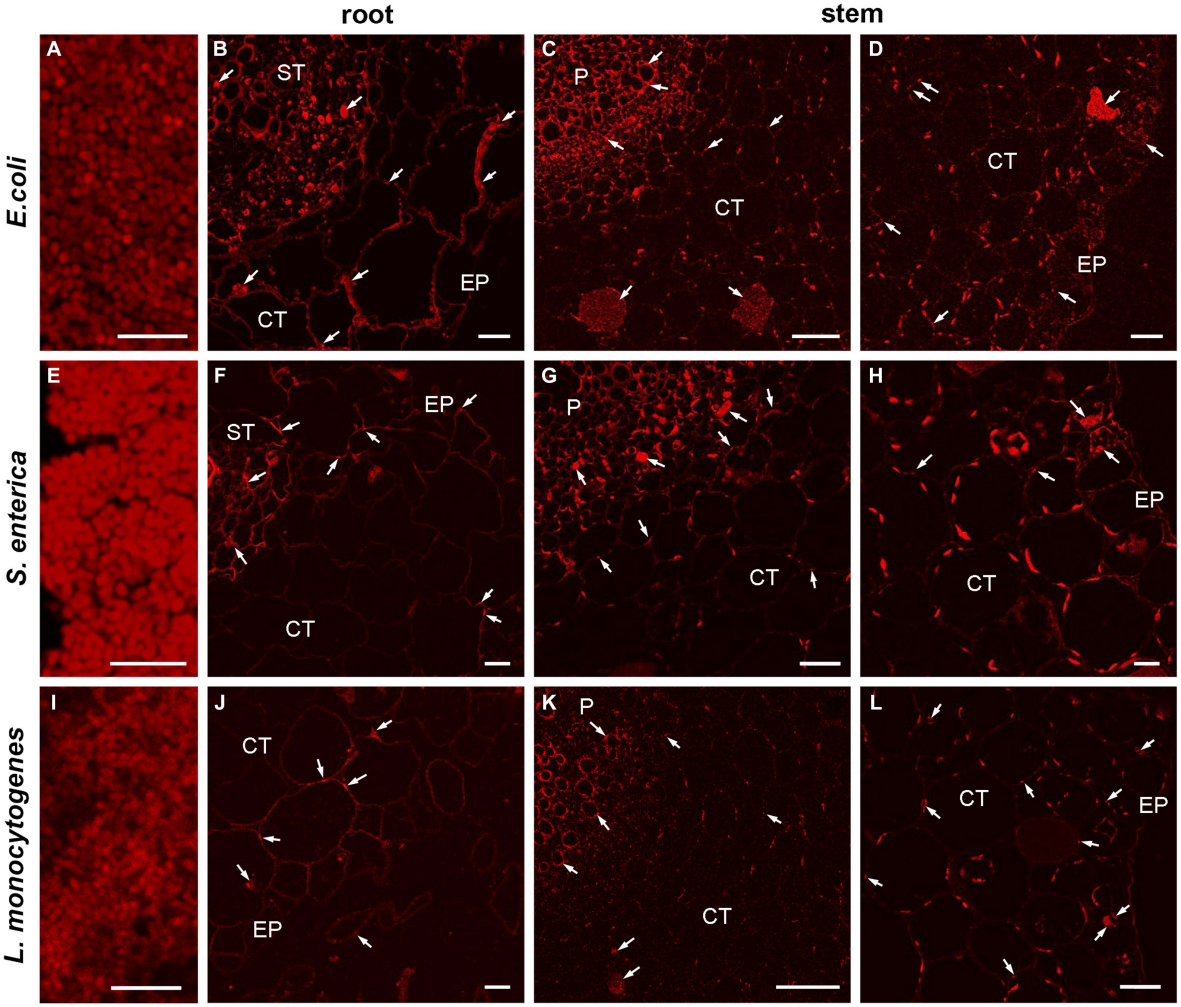
CLSM (confocal laser scanning microscopy) analysis of bacterial (*E. coli*, *L. monocytogenes*, *S. enterica*) colonization pattern of *R. sativus* 1-week seedling root and shoot detected by FISH. The representative images of the positive control of FISH reaction, bars 5 μm **(A,E,I)**, the localization of bacteria in root and in shoot, bars 20 μm **(B,D,G,L)**, bars 10 μm **(F,H,J)** and bars 50 μm **(C,K)**. The arrows indicate the presence of bacteria in analyzed organs: in the extracellular matrix or inside the cells (arrows). The autofluorescence of the chloroplasts are visible **(C,D,G,H,K,L)**, EP, epidermis; CT, cortex tissue; ST, stele tissue; P, pith.

To verify and visualize the bacterial colonization pattern of *R. sativus* roots, stems and leaves we used a *E. coli*, *L. monocytogenes* and *S. enterica* specific sequences targeting the 16S rRNA as specific probes to FISH technique. At first, we optimized the FISH protocol for embedded bacteria detection as positive control of reactions (Figures 8A,E,I) and next we localized the bacteria in semithin sections of inoculated one-week and two-week-old plants. FISH-CLSM analysis confirmed *in situ* presence of all bacteria populations in all organs (roots Figures 8B,F,J and stems Figures 8C,D,G,H,K,L). The fluorescence spots indicating the presence of bacteria were mainly visible in the extracellular matrix of all cell layers of roots, stems and leaves. We also detected bacteria in some the individual cells (for example, Figures 8C,D,G). Similarly, the strong autofluorescence of chloroplasts in leaves completely made impossible the precise localization of bacteria in the apoplast or inside the cells (not shown). Therefore, in this variant of organs further studies of intracellular presence of bacteria at the ultrastructure level in electron microscopy are necessary. In all the negative control reactions no FISH signals were observed. In an uninoculated *R. sativus* 1-week seedling root and shoot only the autofluorescence of cell walls and the chloroplasts are visible (Supplementary Figure S2).

## Discussion

Plants are an attractive niche for many microorganisms, including bacteria, fungi, archaea, viruses, and algae (Truong et al., 2021). Many studies have examined the colonization of various vegetables, e.g., tomatoes, lettuce, cucumbers, and parsley, by individual HPMOs; however, in the case of red radish, there is a lack of comparative studies regarding the interaction of radish with different microbes, including Gram-negative and Gram-positive bacteria (Szymczak et al., 2014; Truong et al., 2021).

In this study, we checked the colonization of *R. sativus* var. *radicula* organs by selected pathogens. The first critical choice we made regarded the method of bacterial inoculation. We decided to inoculate the plant seeds directly by short-term incubation in OD-adjusted HPMO suspensions. Seed inoculation is one of the most effective methods of bacterial delivery to plants (O’Callaghan, 2016; Luna-Guevara et al., 2019). Numerous studies have shown that vegetable contamination by HPMOs can originate from pathogen inoculation into the soil, substrate or water used for plant watering (Islam et al., 2004a,b; Jechalke et al., 2019). Our results, based on culture-dependent and culture-independent methods, revealed the ability of all tested HPMOs, including *E. coli*, *S. enterica*, *L. monocytogenes* and *B. cereus,* to colonize one- and two-week-old radish. Torres et al. (2005) stressed that adhesion is the most important step in the colonization of plants by inoculated pathogens. All studied pathogens are characterized by high adhesion and biofilm formation capacity (Gorski et al., 2003; Yaron and Römling, 2014; Antequera-Gómez et al., 2019; Elpers et al., 2020; Lin et al., 2022). In the adhesion process, an important role is assigned to motility and chemotaxis due to flagellar rotation ability and the presence of the lipopolysaccharide (LPS) layer (Elpers et al., 2020). Liu et al. (2005) confirmed the ability of *S. enterica* and enterohemorrhagic *E. coli* (EHEC) to attach to vegetable seeds. Gorski et al. (2003) revealed high adherence of *L. monocytogenes* to radish slices, and the highest ability was observed in the range of temperatures (20 and 30°C) corresponding to the conditions in which we incubated the seeds in the bacterial suspension (26°C).

The next step of plant colonization after the adhesion of pathogens to the seed surface is their translocation to internal tissues (Melotto et al., 2014). Many scientists have reported a more intensive colonization of internal plant tissues after seed inoculation compared to seedling inoculation (Schoeller et al., 2002; Kim et al., 2018). It is associated with the occurrence of cracks during root development. The cracks are potential places for pathogens to enter the plant. Using these natural openings, bacterial invaders may slow the immune system reaction or even evade plant immune responses (Truong et al., 2021). Our studies showed a high level of one-week *R. sativus* tissue colonization by the tested HPMOs (up to approximately 10^3^ and 10^4^ CFU/g dry weight of shoots and leaves, respectively), which corresponds to the abundance of endophytes observed in plants growing under natural conditions (Compant et al., 2021). A rapid increase in the level of plant colonization by *Salmonella* and *E. coli* O157:H7, reaching from 0.1 log CFU/g to as high as 6 log units, was observed under sprouting conditions (Howard and Hutcheson, 2003). Schoeller et al. (2002) determined the growth and survival capacity of alfalfa sprouts treated with *L. monocytogenes*. In this investigation, plants showed a drastic increase in the density of pathogenic bacteria 1 day postinoculation of seeds. In the same study, the inoculation of sprouted seeds with *L. monocytogenes* performed later (on Day 5) did not result in such an increase (2002). Similarly, Kim et al. (2018) compared the growth dynamics of the *S. enterica* population after inoculation of alfalfa seeds or fully germinated sprouts. The authors found a higher number of pathogens in the case of seed inoculation. Undoubtedly, the choice of inoculation method (in our case, direct seed inoculation) has a key impact on the level of internal plant tissue colonization by bacteria.

In nature, a higher number of bacteria is observed in the roots (from 10^5^ to 10^7^ cultivable bacteria per gram) than in the aboveground parts (10^3^–10^4^) (Compant et al., 2021). Our results for the uninoculated variant, where only endophytes were present inside the plants, showed a high density of bacteria in all radish organs following the trend of roots (ap. 10^4.5^) > shoots > leaves (ap. 10^3^) in the first week after sowing the seeds. Seeds of most plant species are not axenic and are characterized by the presence of bacteria and/or fungi (Truyens et al., 2015; Pitzschke, 2018). We also observed morphologically different bacterial colonies during germination of surface-sterilized radish seeds. Schoeller et al. (2002) observed a drastic increase in the total number of bacteria after 24 h of sprout development from uninoculated seeds (from *ca.* 3.5 log CFU/g to *ca.* 8.0 log CFU/g). The longer cultivation time was associated with a more equal distribution of bacteria in shoots (ap. 10^3.7^) and roots (ap. 10^3.7^) of two-week-old *R. sativus*, while leaves were characterized by the highest density of bacteria (10^4^). It is possible that the presence of compounds with antimicrobial effects in radish roots may cause the movement of bacteria toward the aboveground parts of plants (Beevi et al., 2009). Moreover, in variants inoculated with HPMOs, the interaction between the tested strains and autochthonous endophytes can significantly shape the total number of endophytic microbes, which in turn may affect the process of plant colonization by HPMO (Truong et al., 2021). The presence of HPMOs significantly decreased the total density of bacteria in the roots of one-week *R. sativus*, while in the shoots and leaves, no effect was noted. The exception was a variant treated with *S. enterica*, where an increase in the total density of bacteria in stems and leaves of the week-old radish was observed.

The results of our research confirmed the ability of the tested pathogens not only to colonize plants but also to move from the roots toward the aerial parts. *In situ* studies using FISH-CLSM revealed the presence of HPMOs primarily in apoplast and some cells of roots, stems and leaves in the one-week-old inoculated seedlings. Penetration ability via apoplast has been observed for many HPMOs including *E. coli* serotype O157:H7 (in case of lettuce and spinach through roots and leaves), *L. monocytogenes* (in romanian lettuce) and *S. enterica* (tomatoes) (Shenoy et al., 2017; Wright et al., 2017; Zarkani and Schikora, 2021). The translocation of multiple *Salmonella* serovars (including S. Javiana, S. Newport, S. Poona and S. Montevideo) to the lower stems of cucumber (3–5 cm) 1 week post inoculation into the root zone was observed by Burris et al. (2020). In the case of tomatoes, 7 days after inoculation, distant migration of *S. enterica* (reaching up to 10 cm) from the soil to the shoots was shown (Zheng et al., 2013). There are also reports confirming the ability of *L. monocytogenes* and *E. coli* to translocate from the root zone to the aboveground part (Quilliam et al., 2012; Hofmann et al., 2014; Standing et al., 2014). It was previously discussed that this phenomenon depends on various factors, including the type of substrate and cultivar, time of inoculation and serotype applied (Hirneisen et al., 2012; Burris et al., 2020; Esmael et al., 2023). In this study, *E. coli* showed the highest capacity to colonize roots and stems of one-week-old plants. Most likely, the short time of *E. coli* multiplication (approximately 20 min) influenced the obtained result (Dewachter et al., 2018). Our observation is in line with results described by Standing et al. (2014). The authors, who compared the average density of internalized *E. coli* O157:H7, *L. monocytogenes*, *S. enterica* subsp. enterica serovar Typhimurium and *S. aureus* in one-week-old lettuce roots and leaves, found the highest abundance of *E. coli* in the plant roots, while leaves were characterized by the highest number of *L. monocytogenes* (2013). Similarly, we also observed that *L. monocytogenes* exhibited the highest ability to translocate along the plant and colonize leaves in both one- and two-week-old plants. Distant translocation of bacteria can be associated with flagellum presence and may occur via plant vasculature (Melotto et al., 2014). Shenoy et al. (2017) demonstrated the high mobility of *L. monocytogenes* in the vascular system after inoculation of romaine lettuce seeds.

The raphanin contained in radish seeds and leaves has an antimicrobial effect against several bacteria, including *E. coli, Pseudomonas pyocyaneus, Salmonella typhi*, *Bacillus subtilis*, *S. aureus, streptococci* and *pneumococci,* as well as *Listeria, Micrococcus, Enterococcus, Lactobacillus* and *Pedicoccus* species (Shukla et al., 2011). Our research showed a significant effect of plant age (one- or two-week-old plants were investigated) on colonization by the tested HPMOs. Selected HPMOs had the ability to colonize all organs of one-week *R. sativus*; however, in the case of two-week-old plants, we noted significant changes. Most likely, the concentration of antimicrobial compounds in leaves and roots of *R. sativus* increases with the age of the plant. Among the tested HPMOs, *S. enterica* was the most sensitive strain, and its presence was detected only in stems of two-week-old *R. sativus*. A previous study considering the impact of *R. sativus* extracts on bacterial growth confirmed the higher sensitivity of *S. enterica* compared to other pathogens (Lim et al., 2019). Lim et al. (2019) indicated that *R. raphanistrum* subsp. *sativus* (radish) extract inhibited the growth of *S. enteritidis* 110, *Cronobacter sakazakii* KCTC 2949, *B. cereus* ATCC 10876, and *Staphylococcus aureus* ATCC 6538, while it showed no effect against *L. monocytogenes* ATCC 51776 and *E. coli* 23,716. The roots of two-week-old plants seemed to be a difficult niche for most bacterial strains compared to stems and leaves, as only spore-forming *B. cereus* was able to survive in that organ (Moteshareie et al., 2022). Among extracts prepared from roots, shoots and leaves of *R. sativus,* the first one showed the greatest antibacterial activity against foodborne pathogens, including *B. subtilis, S. aureus, Staphylococcus epidermidis, Enterococcus faecalis, Salmonella typhimurium, Enterobacter aerogenes, Enterobacter cloacae,* and *E. coli* (Beevi et al., 2009). Moreover, Shukla et al. (2011) checked the effect of radish root juice on *Klebsiella pneumoniae*, *Staphylococcus aureus, Pseudomonas aeruginosa*, *Enterococcus faecalis* and *E. coli* growth. The authors observed a greater antibacterial effect on gram-negative than gram-positive bacteria, which is in line with our results showing that gram-negative strains avoided the colonization of radish roots (Shukla et al., 2011).

A plant is a good alternative host for HPMOs, and its colonization can be crucial for the survival of pathogens, first, because the plant provides a refuge for bacteria when it enters the soil, and second, the presence in the plant gives the pathogen a chance for returning to herbivorous and omnivorous hosts (Brandl et al., 2013). Rhizosphere and endophytic microorganisms significantly influence the growth of the host plant (Szymańska et al., 2016). Moreover, HPMOs are also considered important regulators of plant productivity (Nautiyal et al., 2010; Afzal et al., 2019). The plant-HPMO interaction is not fully understood, but it has been indicated that plants can recognize human enteric pathogens and activate basic defense signaling pathways (Brandl et al., 2013). The results of our research suggested that in the first week, the presence of HPMOs may cause a pronounced plant stress reaction, which was manifested, among the others, by the decrease in plant fresh weight. After 2 weeks, the plant response was less noticeable, which may indicate that the plants are adapting to the stress caused by the presence of pathogens. Jechalke et al. (2019) showed that lettuce responded to the presence of *S. typhimurium* 14,028 s through upregulation of genes associated with the stress response and genes related to the plant immune response. Among the pathogens studied by us, *L. monocytogenes* exerted the most unfavorable effect on the radish growth parameters. Similar to our results, Klerks et al. (2007) observed the inhibition of lettuce seedling growth and their biomass reduction after inoculation with *Salmonella* Dublin. Furthermore, the negative impact of HPMOs on many different plants, e.g., tomato, romaine lettuce, and *Medicago truncatula,* was previously found (Jayaraman et al., 2014; Deering et al., 2015; Simko et al., 2015). Furthermore, the symptoms noticed in plants after HPMO inoculation included chlorosis, wilting, tissue necrosis or root growth inhibition, which was also observed by researchers (Klerks et al., 2007; Schikora et al., 2008; Berger et al., 2011).

In summary, we conclude that all tested HPMOs (*E. coli, S. enterica, L. monocytogenes* and *B. cereus*) have the ability to colonize radish no later than in the first week of its growth. *E. coli* and *L. monocytogenes* were characterized by the highest ability to migrate along the plant (from roots, through shoot to leaves) and to colonize the above-ground plant organs. Plant age significantly influenced the distribution of HPMOs in *R. sativus* organs. The tested HPMOs did not colonize the two-week-old radish roots (with the exception of *B. cereus*, which most likely survived due to the formation of spores). Only *L. monocytogenes* and *E. coli* preferred to colonize the leaves of two-week-old radish. Limited colonization of roots and leaves of two-week-old *R. sativus* by HPMOs could be related to the presence of compounds with antibacterial properties in these radish organs. On the other hand, the presence of pathogens in plant organs inhibits the growth of radish, which is manifested by a decrease in growth parameters after the first week. Nevertheless, *R. sativus* can adapt to the presence of pathogens, causing no further decrease in the growth rate of two-week-old plants.

## Data availability statement

The raw data supporting the conclusions of this article will be made available by the authors, without undue reservation.

## Author contributions

SS: Conceptualization, Data curation, Formal analysis, Funding acquisition, Investigation, Methodology, Project administration, Resources, Visualization, Writing – original draft. ED-S: Investigation, Methodology, Supervision, Visualization, Writing – review & editing. MS: Investigation, Methodology, Writing – review & editing. KN: Investigation, Methodology, Visualization, Writing – review & editing. JM: Investigation, Writing – review & editing. KH: Supervision, Writing – review & editing.

## Supplementary material

Supplementary Figure S1 Density of cultivable endophytes (total density of endophytes; (**A-C**) and human pathogenic microorganism (HPMO, including *L. monocytogenes* PCM 2191 – L.m., *B. cereus* PCM 1948 – B.c., *E. coli* PCM 2561 – E.c. and *S. enterica* subsp. *enterica* PCM 2565 – S.e.; (**D-F**) in the roots, stems and leaves of *R. sativus* after one and two weeks of plant cultivation. Significant differences (*p* < 0.05, one-way ANOVA with Newman-Keuls post hoc comparisons) between treatments (Ctr, control, noninoculated; *L.m*., inoculated with *L. monocytogenes* PCM 2191; *B.c*., inoculated with *B. cereus* PCM 1948; *E.c*., inoculated with *E. coli* PCM 2561 and *S.e*., inoculated with *S. enterica* subsp. *enterica* PCM 2565) at each *R. sativus* organ are denoted by different letters (including capital and small letters in the case of one- and two-week-old plants, respectively); differences between values obtained for each variant of experiment (*Ctr*, *L.m*., *B.c*., *E.c*. and *S.e*.) in the case of tree tested organs are marked with different marks (*). The mean ± standard deviation are presented (*n* = 3).

Supplementary Figure S2 The representative CLSM (confocal laser scanning microscopy) images of the negative control of FISH reaction of bacterial (*E. coli, S. enterica, L. monocytogenes*) colonization of an uninoculated *R. sativus* 1-week seedling root and shoot, bars 50 µm (**A, B, D, E, G, H**) and bars 30 µm (**C, F, I**). Only the autofluorescence of cell walls and the chloroplasts are visible, EP – epidermis, CT – cortex tissue, ST – stele tissue, P – pith.
