# Supplementary figures and images for: Colonization of Raphanus sativus by human pathogenic microorganisms
Source: Front Microbiol. 2024 Feb 15;15:1296372. doi: 10.3389/fmicb.2024.1296372 (PMC10902717; doi:10.3389/fmicb.2024.1296372)

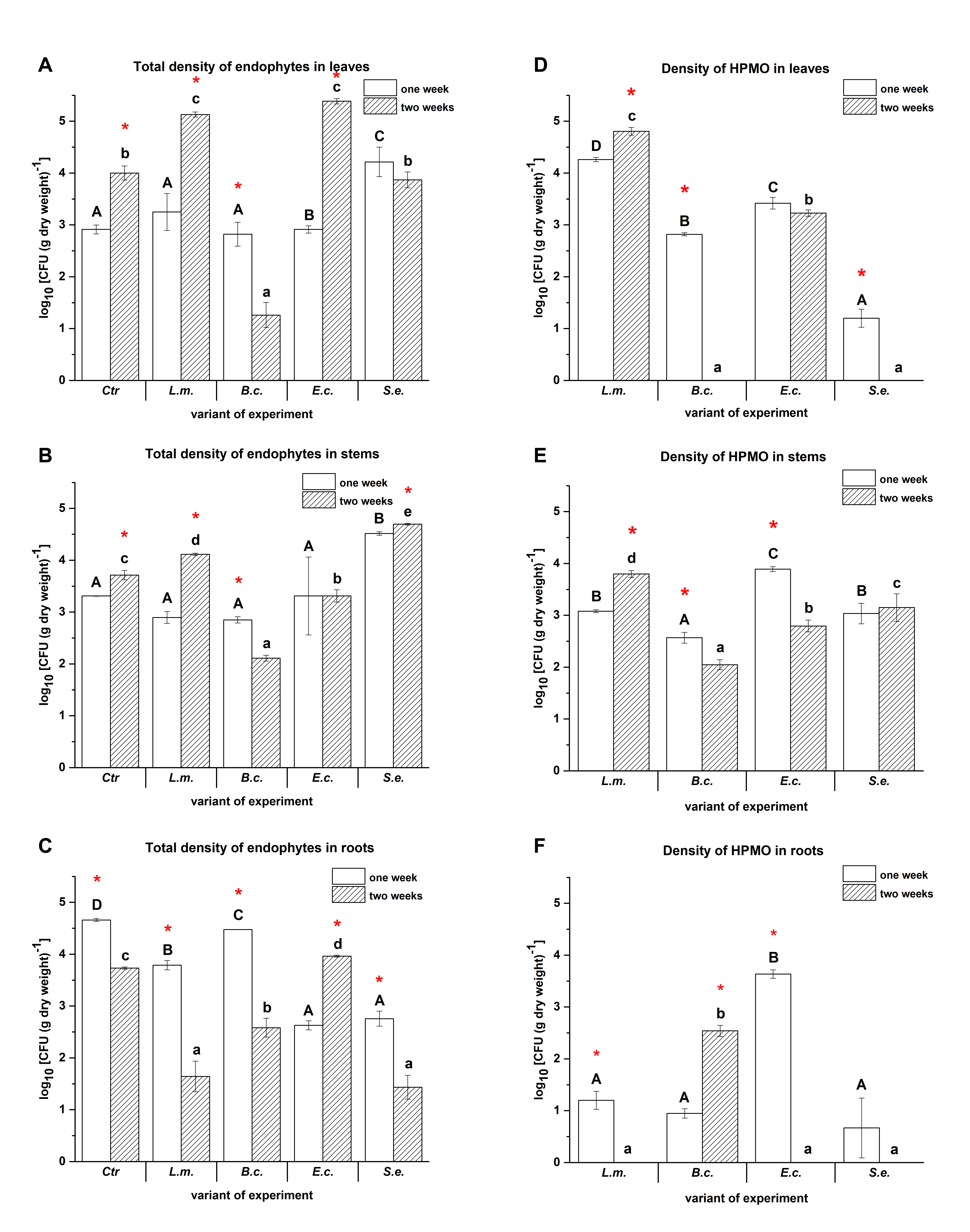

Supplement: Supplementary Figure S1 — Density of cultivable endophytes (total density of endophytes; (A-C) and human pathogenic microorganism (HPMO, including L. monocytogenes PCM 2191 – L.m., B. cereus PCM 1948 – B.c., E. coli PCM 2561 – E.c. and S. enterica subsp. enterica PCM 2565 – S.e.; (D-F) in the roots, stems and leaves of R. sativus after one and two weeks of plant cultivation. Significant differences (p < 0.05, one-way ANOVA with Newman-Keuls post hoc comparisons) between treatments (Ctr, control, noninoculated; L.m., inoculated with L. monocytogenes PCM 2191; B.c., inoculated with B. cereus PCM 1948; E.c., inoculated with E. coli PCM 2561 and S.e., inoculated with S. enterica subsp. enterica PCM 2565) at each R. sativus organ are denoted by different letters (including capital and small letters in the case of one- and two-week-old plants, respectively); differences between values obtained for each variant of experiment (Ctr, L.m., B.c., E.c. and S.e.) in the case of tree tested organs are marked with different marks (*). The mean ± standard deviation are presented (n = 3). [file Image_1.TIF]

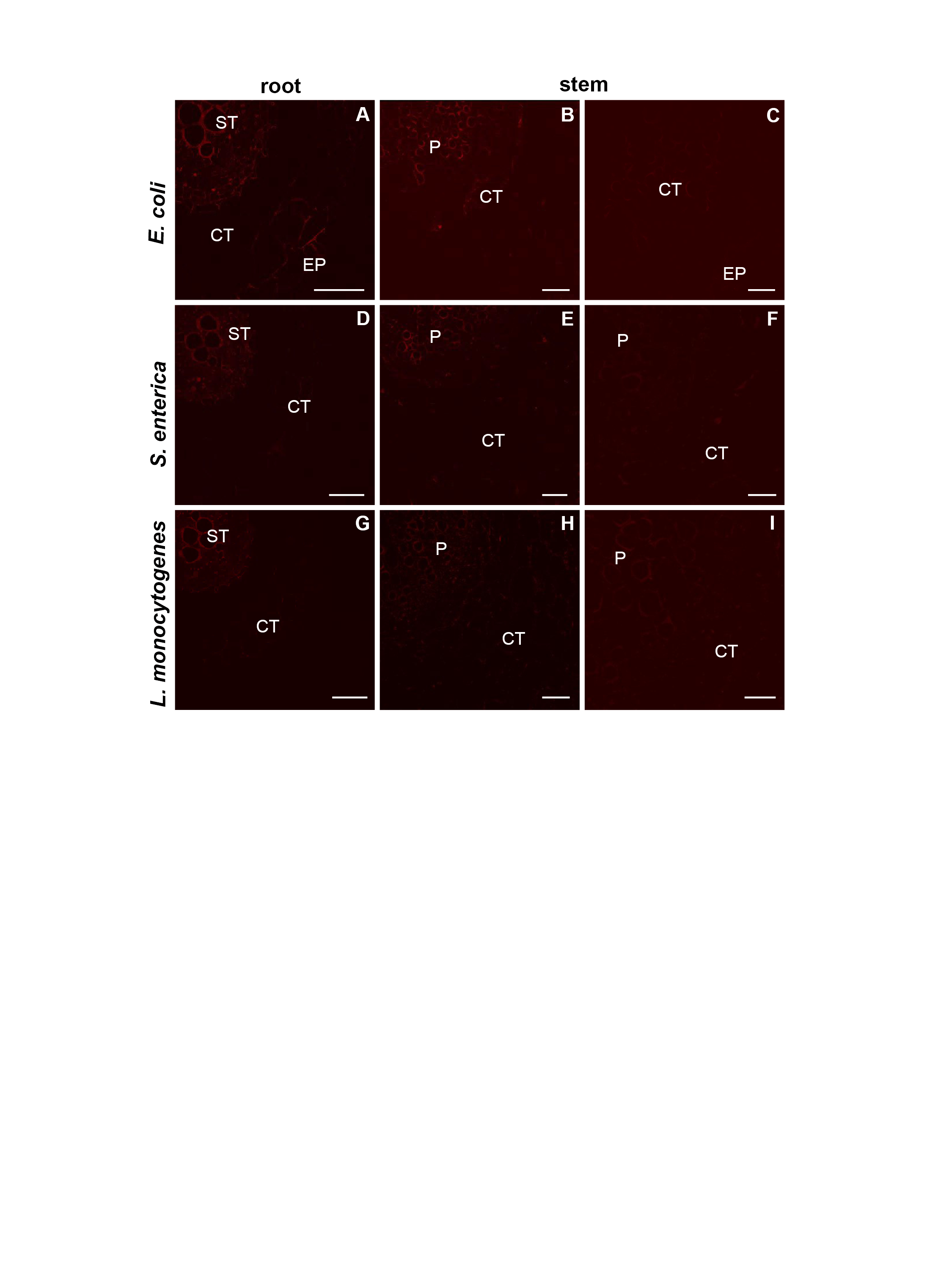

Supplement: Supplementary Figure S2 — The representative CLSM (confocal laser scanning microscopy) images of the negative control of FISH reaction of bacterial (E. coli, S. enterica, L. monocytogenes) colonization of an uninoculated R. sativus 1-week seedling root and shoot, bars 50 µm (A, B, D, E, G, H) and bars 30 µm (C, F, I). Only the autofluorescence of cell walls and the chloroplasts are visible, EP – epidermis, CT – cortex tissue, ST – stele tissue, P – pith. [file Image_2.TIF]
